# Urinary Proteomics Identifying Novel Biomarkers for the Diagnosis and Phenotyping of Carotid Artery Stenosis

**DOI:** 10.3389/fmolb.2021.714706

**Published:** 2021-08-10

**Authors:** Wei Wang, Jianqiang Wu, Peng Liu, Xiaoyue Tang, Haiyu Pang, Ting Xie, Fang Xu, Jiang Shao, Yuexin Chen, Bao Liu, Yuehong Zheng

**Affiliations:** ^1^ State Key Laboratory of Complex Severe and Rare Disease, Department of Vascular Surgery, Peking Union Medical College Hospital, Chinese Academy of Medical Sciences and Peking Union Medical College, Beijing, China; ^2^ State Key Laboratory of Complex Severe and Rare Diseases, Medical Research Center, Peking Union Medical College Hospital, Chinese Academy of Medical Sciences and Peking Union Medical College, Beijing, China

**Keywords:** carotid artery stenosis, stroke, proteomics, biomarkers, urine

## Abstract

**Background:** Carotid artery stenosis (CAS) is caused by the formation of atherosclerotic plaques inside the arterial wall and accounts for 20–30% of all strokes. The development of an early, noninvasive diagnostic method and the identification of high-risk patients for ischemic stroke is essential to the management of CAS in clinical practice.

**Methods:** We used the data-independent acquisition (DIA) technique to conduct a urinary proteomic study in patients with CAS and healthy controls. We identified the potential diagnosis and risk stratification biomarkers of CAS. And Ingenuity pathway analysis was used for functional annotation of differentially expressed proteins (DEPs). Furthermore, receiver operating characteristic (ROC) analysis was performed to evaluate the diagnostic values of DEPs.

**Results:** A total of 194 DEPs were identified between CAS patients and healthy controls by DIA quantification. The bioinformatics analysis showed that these DEPs were correlated with the pathogenesis of CAS. We further identified 32 DEPs in symptomatic CAS compared to asymptomatic CAS, and biological function analysis revealed that these proteins are mainly related to immune/inflammatory pathways. Finally, a biomarker panel of six proteins (ACP2, PLD3, HLA-C, GGH, CALML3, and IL2RB) exhibited potential diagnostic value in CAS and good discriminative power for differentiating symptomatic and asymptomatic CAS with high sensitivity and specificity.

**Conclusions:** Our study identified novel potential urinary biomarkers for noninvasive early screening and risk stratification of CAS.

## Introduction

Carotid artery stenosis (CAS) is characterized by the formation of atherosclerotic plaques in the arterial wall and accounts for approximately 20–30% of all strokes (Wijeratne et al., 2020). Stroke is the second leading cause of mortality and a leading cause of adult disability worldwide, making prevention of stroke one of the most important priorities in healthcare (Feigin et al., 2014; Banerjee and Chimowitz, 2017; Feigin et al., 2018). Atherosclerosis (AS) is central to the pathogenesis of CAS. AS usually occur at sites of blood flow disturbance. And it was thought to be triggered by subendothelial retention of cholesterol-containing lipoproteins, flow-mediated inflammation within the endothelial cells, and inflammatory cell infiltration (Hansson et al., 2015). The risk factors for CAS are associated with traditional atherosclerotic risk factors such as hypertension, diabetes, dyslipidemia, smoking, and advanced age (Dharmakidari et al., 2017; Wijeratne et al., 2020).

The progression of CAS is usually insidious, and patients may remain asymptomatic until the appearance of transient ischemic attacks (TIA) or stroke symptoms. Thus, early diagnosis of CAS is essential for the management of CAS. However, there is currently no specific and sensitive biomarker for the early, rapid, noninvasive diagnosis of CAS. CAS can be clinically classified into symptomatic and asymptomatic CAS. Patients with symptomatic carotid stenosis carry a higher stroke risk than asymptomatic patients, and the management of asymptomatic and symptomatic CAS is also different (Hackam, 2016; Dharmakidari et al., 2017; Gavin et al., 2018). Characterizing symptomatic CAS could contribute to the early prediction, risk stratification and early intervention of stroke or TIA.

In the last decade, mass spectrometry (MS)-based proteomic analysis has revolutionized the search for disease biomarkers by detecting thousands of proteins from various sample sources. The intrinsic properties of urine make it an attractive source of biomarkers for clinical proteomic studies. Because of the advantages of the noninvasive nature, simple collection and stable condition of urine, urinary proteomics has been widely used in screening, diagnosis or monitoring of diseases such as cardiovascular disease, kidney disease, and cancer (Röthlisberger and Pedroza-Diaz, 2017; Chen et al., 2018; Erozenci et al., 2019; Ferrari et al., 2019). However, the urine protein profiles of patients with CAS are still unknown and might provide potential biomarkers for disease diagnosis and risk stratification.

In this study, we used the DIA technique to perform urinary proteomics and explore the urinary protein profile in patients with CAS. Bioinformatics analysis of differentially expressed proteins (DEPs) was further performed to validate the association between DEPs and the pathophysiological process of CAS. We identified several urinary proteins that had the potential to discriminate CAS patients from healthy controls (HCs) and distinguish symptomatic patients from asymptomatic CAS patients. This study may provide a noninvasive and effective method for CAS diagnosis and risk stratification.

## Materials and Methods

### Patients and Sample Collection

The urine samples used for proteomic analysis were obtained from consecutive patients with CAS before they underwent surgery at Peking Union Medical College Hospital from January 2020 to January 2021, and urine samples from matched HCs were collected during the same period. The study was approved by the Ethics Committee of Peking Union Medical College Hospital (No. JS-2629), and all patients and volunteers provided written consent and assent. All experiments conformed to the Declaration of Helsinki.

Patients were considered symptomatic if they had neurologic symptoms that were sudden in onset and referable to the appropriate carotid artery distribution, including one or more TIA characterized by focal neurologic dysfunction or transient monocular blindness or one or more minor (nondisabling) ischemic strokes in the previous 180 days (Brott et al., 2016). Ultrasonographic characteristics of the plaques echolucency in CAS patients were based on the modified version of the Gray–Weale classification (Gray-Weale et al., 1988). Plaque echolucency was classified as: hyperechogenic plaque, mixed echogenic plaques and hypoechogenic plaque (Silvestrini et al., 2013). Eligibility criteria for symptomatic patients included extracranial stenosis of 50% or more of the diameter of the artery on angiography (based on North American Symptomatic Carotid Endarterectomy Trial [NASCET] criteria), 70% or more on ultrasonography, or 70% or more on computed tomography angiography (CTA) or magnetic resonance angiography (MRA) if the stenosis on ultrasonography was 50–69%. The eligibility criteria of asymptomatic patients included extracranial stenosis of 60% or more on angiography, 70% or more on ultrasonography, or 80% or more on CTA or MRA if the stenosis on ultrasonography was 50–69%. The control group consisted of volunteers without significant differences in sex, age, hypertension and diabetes from the patient groups, and all control participants were excluded from a diagnosis of CAS by ultrasonography of the carotid artery. The exclusion criteria were as follows: 1) surgery in the last 6 months; 2) the presence of systemic inflammatory or infectious diseases such as hypothyroidism and Takayasu arteritis; 3) cancer; 4) severe peripheral artery disease; 5) History of chronic heart failure (Cardiac Function Standard of the New York Heart Association: class II - IV) (Hou et al., 2014) and renal insufficiency; and 6) proteinuria, hematuria or pyuria in routine urine test.

Midstream urine samples were collected from patients and HCs in the morning and were immediately centrifuged at 4,500rpm at 4°C for 15 min to remove the cellular components and debris, and the supernatants were stored at −80°C for further analysis.

### Sample Preparation

The urine sample was processed by a filter-aided sample preparation (FASP) method as previously described (Wiśniewski et al., 2009). The proteins were reduced with 20 mM DTT at 100°C for 10 min and alkylated with 50 mM iodoacetamide (IAM) at room temperature for 45 min in the dark. Next, 6X precooled-acetone precipitation was used to extract proteins. Then, the protein precipitates were redissolved with 20 mM Tris and transferred to a 30-kD cutoff ultrafiltration unit. After washing the sample with 20 mM Tris three times, the proteins were digested with trypsin (1:50) at 37°C overnight. Finally, the peptide mixtures were collected by centrifugation and then quantified using the BCA method.

### Offline HPLC Separation

An equal amount of digested peptides of each sample were pooled to generate a mixed sample for generating a spectral library for DIA analysis. The mixed sample was dried and resuspended in Buffer A (90% ACN, 10% H_2_O, with 0.1% FA and 10 mM ammonium formate). Then the sample was separated by a high pH reversed-phase liquid chromatography column (4.6 mm × 250 mm, Xbridge C18, 3 mm). After equilibration of the column, the sample was loaded onto the column and eluted with 5–70% buffer B (H_2_O with 0.1% FA; flow rate = 1 ml/min). The eluted peptides were collected at one fraction per minute. We concatenated the 60 collected fractions into 20 fractions (concatenation scheme: 1 + 21 + 41, 2 + 22 + 42, etc). The 20 dried fractions were resuspended in 0.1% FA for subsequent LC-MS/MS analysis.

### DIA Proteomics Analysis

LC-MS/MS analysis was performed by an Orbitrap QE HF (Thermo Scientific, Germany) coupled with an EASY-nLC 1200 UHPLC system. For spectral library establishment, 6 μl of each of 20 fractions was analyzed by data-dependent acquisition (DDA) analysis. The full scan was performed from 350–1,500 m/z. The autogain control (AGC) was set 1e6, and the max injection time was 50 ms. Precursors were screened with a charge state of +2 to +6 and within a dynamic exclusion duration of 30 s. The isolation of precursors was by the quadrupole with a 1.6 m/z isolation window. The most intense ions per survey scan were used for fragmentation. The resulting fragments were analyzed in the Orbitrap analyzer with a resolution of 60,000. The normalized collision energy for HCD-MS_2_ experiments was 32% with the AGC target of 5e4. The maximum injection time was 30 ms, and the DDA cycle was 3 s.

For DIA analysis, the LC settings were the same as in the DDA experiment to maintain retention time stability. The digested peptides of clinical samples were dissolved in 0.1% FA and loaded on a trap column. The eluent was transferred to a reversed-phase analytical column (75 μm × 500 mm, 2 μm, MONOTECH, Kyoto, Japan). The eluted gradient was 5–30% buffer B (0.1% formic acid in 99.9% acetonitrile; flow rate of 0.6 ml/min) for 60 min. Moreover, an iRT kit (Biognosys, Schlieren, Switzerland) was added in all samples to make retention time alignments among samples (Escher et al., 2012). The MS parameters were set as follows: the full scan was performed with a resolution of 120,000 and in the range of 350–1,500 m/z; the cycle time was 3 s; the AGC was 3e6, and the injection time was under 100 ms; charge state screening was performed by precursors with a +2 to +6 charge state; and the dynamic exclusion duration was 10 s. The precursor ion number was equalized in each isolation window according to the precursor m/z distribution of the pooled sample. During the DIA analysis, a mixed sample was inserted after every ten samples for quality control (QC).

### Proteomics Data Analysis

The raw MS data from DDA analysis were searched using Proteome Discoverer software (version 2.1; Thermo Fisher, Waltham, MA, United States) with SEQUEST HT against the SwissProt human database appended with the iRT fusion protein sequence. The parent ion tolerance was set at 10 ppm, and the fragment ion mass tolerance was set to 0.05 Da. A maximum of two missed cleavage sites in the trypsin digestion was allowed. Carbamidomethylation of cysteines was set as a fixed modification, and the oxidation of methionine was considered a variable modification. The false discovery rate (FDR) of protein identifications was accepted less than 1.0%. Then, the results were imported to Spectronaut Pulsar X (Biognosys AG, Switzerland) software to generate the spectra library.

Then, all the raw DIA files were imported into Spectronaut Pulsar X as default settings. The optimal XIC extraction window was determined according to the iRT calibration strategy. The mass tolerance strategy was set to dynamic based on the extensive mass calibration. The cross-run normalization was set to the local normalization based on the local regression. The sum peak areas of the respective fragment ions in MS_2_ were used for the quantification of peptide intensities. The missing values of protein abundance were filled with the KNN method.

### GO and Ingenuity Pathway Analysis (IPA)

DEPs were assigned to their gene symbols according to the PANTHER database (http://www.pantherdb.org/) to perform GO analysis (Mi et al., 2017). The urine proteins were classified based on their molecular function, biological process, and cellular component. For IPA analysis, all DEPs were included for pathway and function analysis. SwissProt accession numbers were used as inputs for Ingenuity Pathway Analysis (version 2.3; Qiagen, CA, United States).

### Statistical Analysis

Data analysis and visualization were conducted by GraphPad Prism (version 8.0.2, GraphPad Software Inc., San Diego, California, United States) and SPSS version 20 (SPSS Inc., IBM, New York, United States). Statistical comparisons between two groups were performed with Student’s t-test (two-tailed), and for comparisons of more than two groups, analysis of variance (ANOVA) was performed. Orthogonal partial least squares discriminant analysis (OPLS-DA) was conducted by SIMCA software (version15.0, Umetrics, Sweden). Receiver operating characteristic (ROC) analysis was performed with the “Biomarker discovery” module in MetaAnalyst 5.0 website (https://www.metaboanalyst.ca/) and MedCalc - version 20.009 (MedCalc Software Ltd.).

## Results

### Clinical Characteristics of Patients With CAS and Study Design

Thirty-five patients with CAS (asymptomatic: 23 patients; symptomatic: 12 patients) and 18 HCs were included in the present study. The clinical characteristics, laboratory values of each subject, ultrasonographic characteristics of the carotid plaques and more common drug usage were recorded and are shown in Table 1. In the matching process, we mainly focused on several potential confounding factor, and healthy controls was matched by the proportion of the age, sex, hypertension and diabetes in CAS group. The distribution of above variables was coincident between CAS group and health control group (*p* > 0.05).

**TABLE 1:** Clinical characteristics of subjects in this study.

Variable	HCs	sCAS	aCAS	*p* value^a^
Number	18	12	23	-
Sex (male)	13/5	10/2	18/5	0.769
Age (years)	65 ± 6	68 ± 5	67 ± 8	0.321
Hypertension	61% (11)	75% (9)	61% (14)	0.672
Diabetes mellitus	44% (8)	50% (6)	39% (9)	0.822
Dyslipidemia	33% (6)	42% (5)	52% (12)	0.478
Coronary artery disease	22% (4)	33% (4)	39% (9)	0.513
Homocysteine	11.3 ± 4.5	17.2 ± 9.9	14.0 ± 6.0	0.066
Uric Acid	379 ± 95	271 ± 102	331 ± 121	0.034^b^
High-sensitivity C-reactive protein	0.89 ± 0.79	0.85 ± 1.71	1.13 ± 1.39	0.744
Total cholesterol	4.38 ± 0.93	3.59 ± 0.72	3.72 ± 0.86	0.028^b^
Triglyceride	1.33 ± 0.67	0.91 ± 0.28	1.21 ± 0.48	0.166
HDL-Cholesterol	1.35 ± 0.27	1.07 ± 0.17	1.09 ± 0.22	0.001^b^
LDL-Cholesterol	2.55 ± 0.75	2.08 ± 0.69	2.10 ± 0.77	0.137
Creatinine	78 ± 11	74 ± 20	75 ± 16	0.712
Hypoechogenic plaque	-	58% (7)	30% (7)	0.153
Acasbose	-	17% (2)	22% (5)	1.000
Metformin	-	17% (2)	17% (4)	1.000
Dihydropyridine calcium antagonists	-	42% (5)	39% (9)	1.000
Renin-angiotensin system inhibitors	-	25% (3)	26% (6)	1.000

HCs, healthy controls; aCAS, asymptomatic CAS; sCAS, symptomatic CAS.

a Comparison of the three groups was performed using the analysis of variance (ANOVA) or Chi-square test.

b *p* value < 0.05 among HCs, sCAS and aCAS are indicated as significant; Dihydropyridine calcium antagonists include nifedipine or amlodipine; Renin-angiotensin system inhibitors include angiotensin converting enzyme inhibitor or angiotensin II receptor blockers.

**FIGURE 1:**
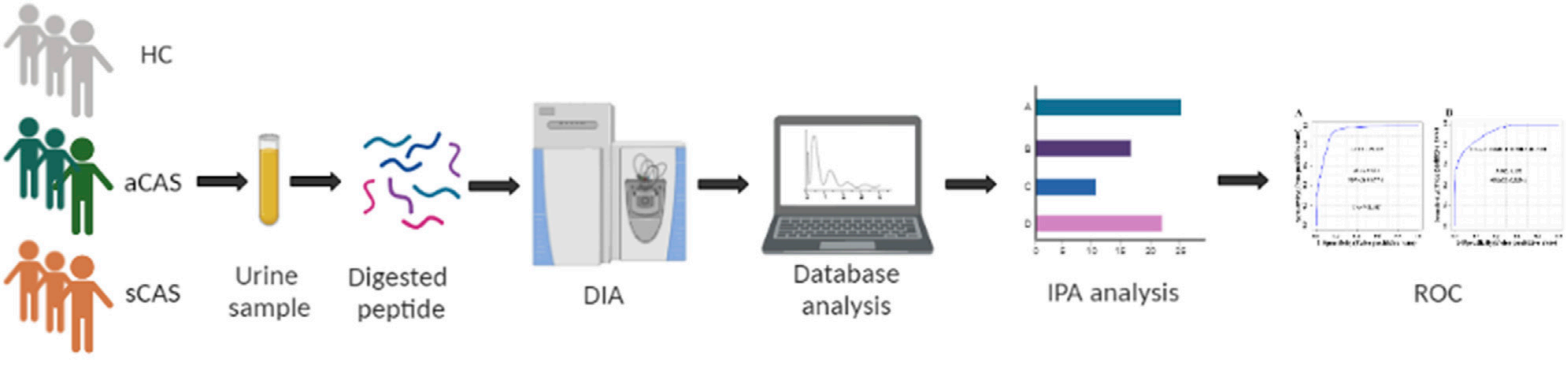
Workflow of urine proteome profile analysis of this study. HCs: healthy controls. aCAS: asymptomatic CAS. sCAS: symptomatic CAS. DIA: data-independent acquisition. ROC: receiver operating characteristic curve.

Urinary proteomes were compared among patients with two subtypes of CAS and HCs. The flow chart of the study design is presented in Figure 1. Urinary proteomes were compared among symptomatic CAS patients, asymptomatic CAS patients and HCs using the DIA technique. The key DEPs were screened, and bioinformatics analysis and ROC analysis were performed.

### Identification of Potential Diagnostic Biomarkers for CAS and Bioinformatics Analysis of DEPs

A mixed sample was used to assess the stability of the results. The correlations of the quantified protein intensities of the QC samples present satisfactory reproducibility (R^2^-values: approximately 0.99, Supplementary Figure S1). Overall, 1,668 proteins were identified in 53 samples with at least two unique peptides (Supplementary Table S1).

**FIGURE 2:**
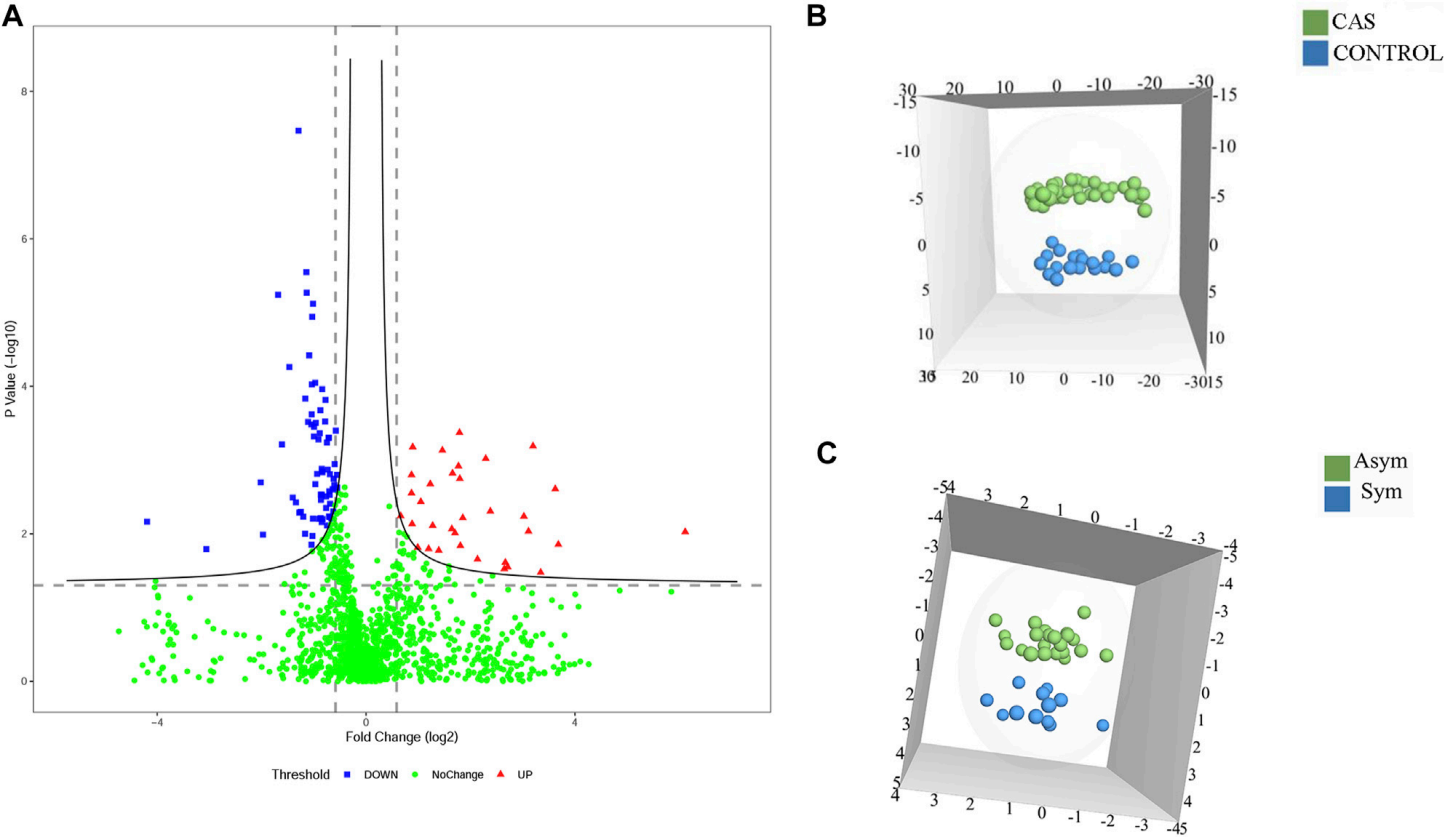
The DEPs among HCs and asymptomatic and symptomatic CAS patients. **(A)** A volcano plot of protein expression in CAS patients compared to HCs; **(B)** The OPLS-DA model between CAS patients and HCs; **(C)**. The OPLS-DA model between asymptomatic and symptomatic CAS patients.

To identify potential diagnostic biomarkers of CAS, urine proteomics analysis was conducted by DIA methods between patients with CAS and HCs. The data showed that 194 DEPs were identified (fold change >1.5; *p* < 0.05), with 131 proteins upregulated and 63 downregulated (Figure 2A; Supplementary Table S2). OPLS-DA was performed based on the DEPs, and the results showed that the HCs group could be separated from the CAS group (Figure 2B).

**FIGURE 3:**
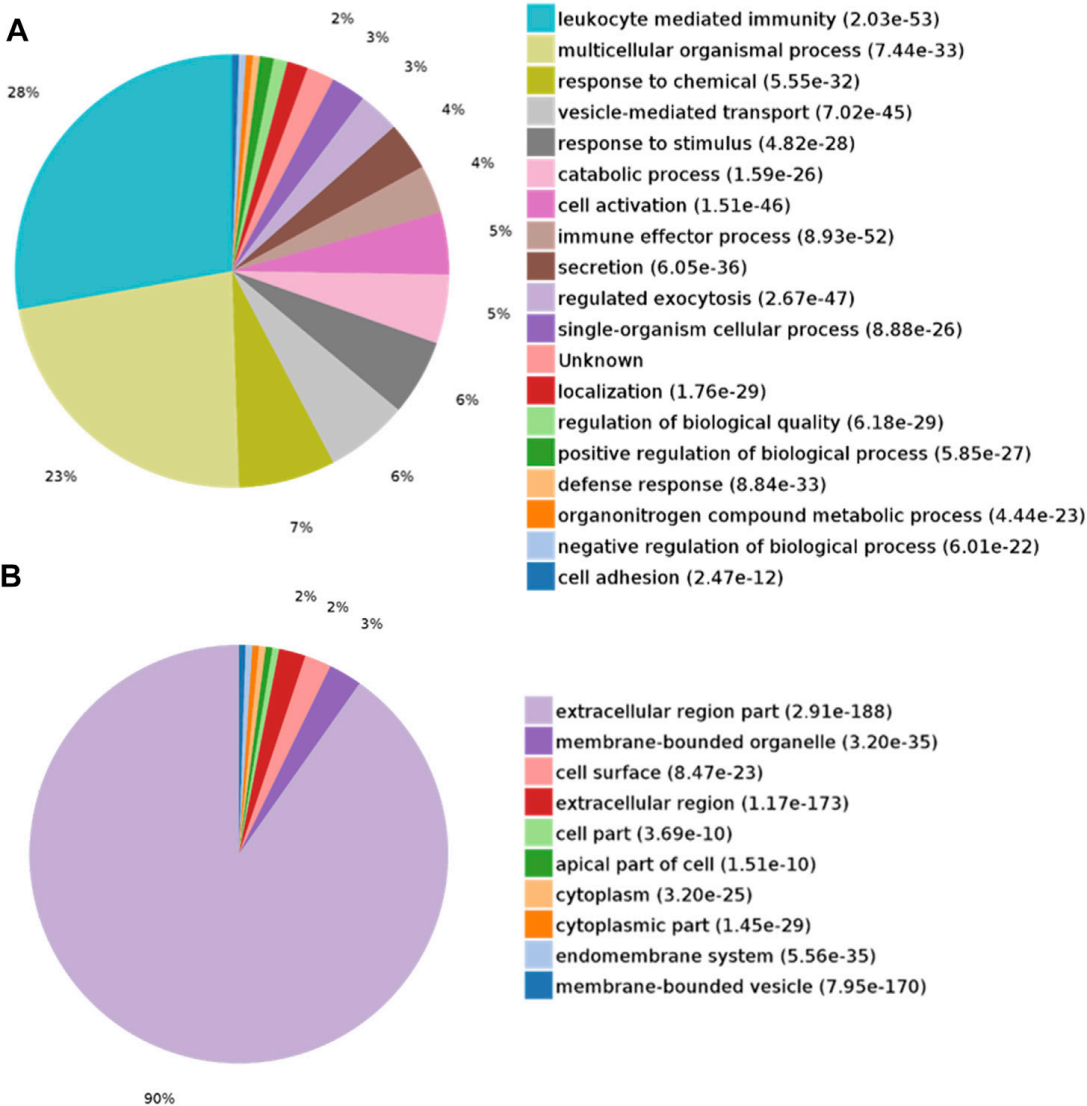
GO analysis of differentially expressed proteins in CAS. **(A)** Biological process; **(B)** Cellular component.

**FIGURE 4:**
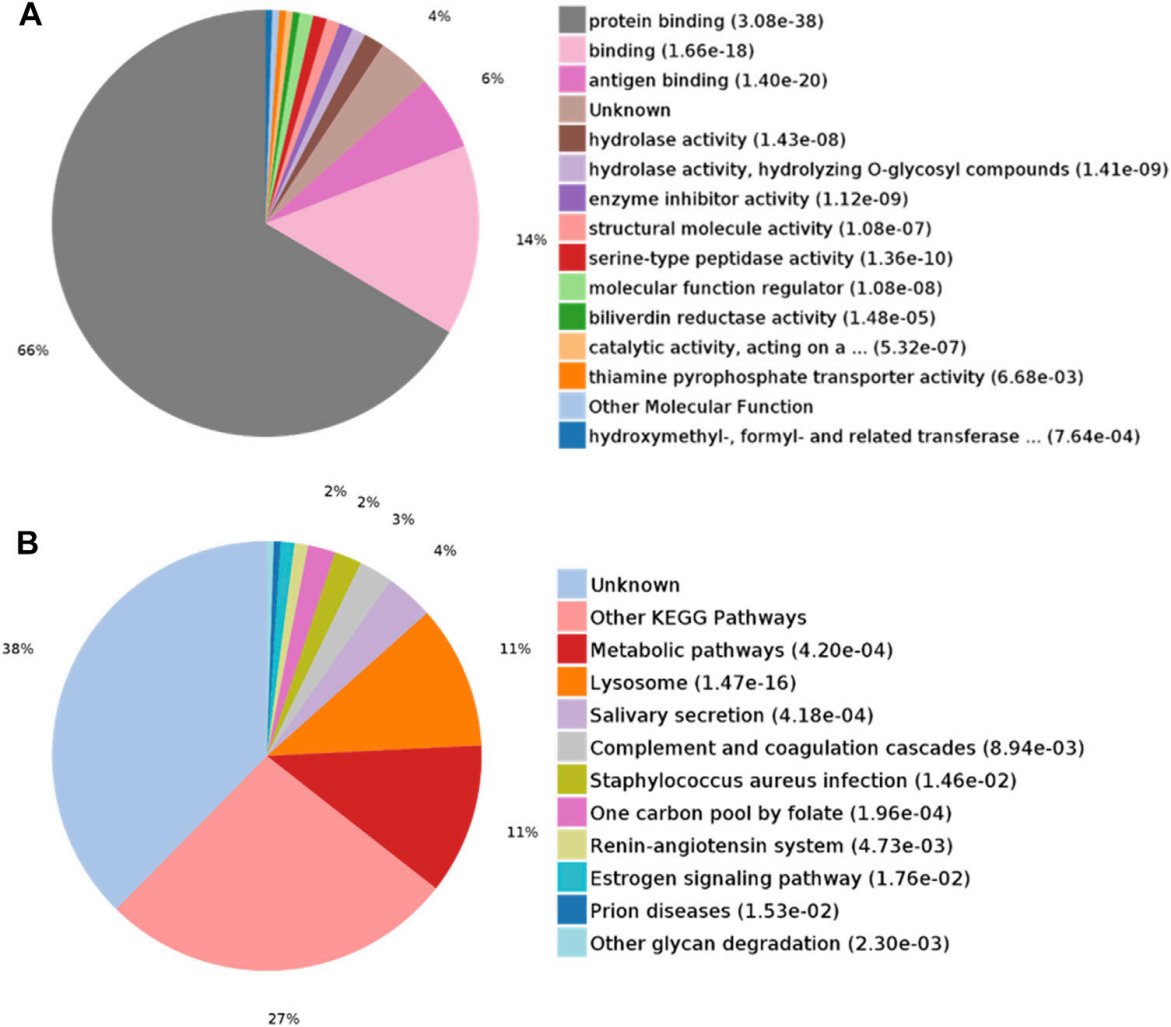
GO analysis of differentially expressed proteins in CAS. **(A)** Molecular function; **(B)** KEGG pathway analysis.

DEPs were searched for the enrichment of GO terms in the PANTHER Classification System. In the biological process category, proteins were mainly enriched in leukocyte-mediated immunity (28%) and multicellular organismal processes (23%) (Figure 3A). In the cellular component category, the extracellular region part (90%) was overrepresented (Figure 3B). In the molecular function category, protein binding (66%) was overrepresented (Figure 4A). According to the Kyoto Encyclopedia of Genes and Genomes (KEGG) pathway analysis, metabolic-related pathways (11%) and lysosome-related pathways (11%) were significantly related to CAS (Figure 4B).

**FIGURE 5:**
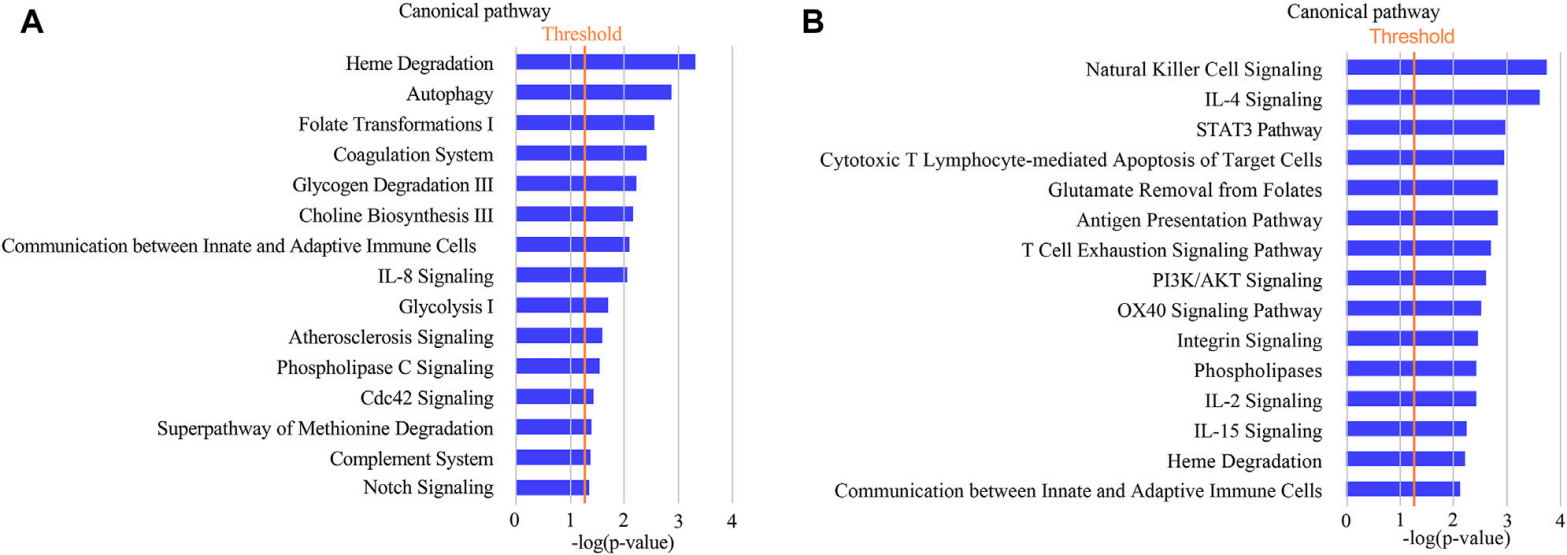
Annotation and functional characterization of differentially expressed proteins. **(A)**: HCs and CAS patients; **(B)**: asymptomatic patients and symptomatic patients with CAS.

IPA analysis was performed to further understand the functional characterization of the DEPs. Canonical pathway analysis revealed that several pathways were significantly enriched in CAS, including heme degradation, autophagy, folate transformation I, coagulation system, glycogen degradation III, choline biosynthesis III, communication between innate and adaptive immune cells, and IL-8 signaling (Figure 5A).

### Identification of Potential Biomarkers of Symptomatic CAS and Bioinformatics Analysis of DEPs

To characterize symptomatic CAS and identify potential biomarkers of high risk of stroke or TIA, proteomics analysis was also performed between symptomatic and asymptomatic patients. There were 32 DEPs (*p*-value < 0.05 and fold change >1.5), including 21 upregulated proteins and 11 downregulated proteins (Supplementary Table S3). OPLS-DA was performed, and the results showed that asymptomatic and symptomatic CAS groups could be separated from each other in the OPLS-DA model (Figure 2C). In addition, 100 permutations were used to validate the OPLS-DA, which showed no overfitting.

IPA analysis showed that the functions of the DEPs were mainly associated with immune/inflammatory pathways (such as natural killer cell signaling, IL-4 signaling, STAT3 pathway, cytotoxic T lymphocyte-mediated apoptosis of target cells, antigen presentation pathway, T cell exhaustion signaling pathway) in symptomatic CAS (Figure 5B). The results suggested that proteins related to immune/inflammatory pathways may play important roles in symptomatic carotid artery stenosis.

To further investigate potential clinical significance of the 32 DEPs, we also evaluated the association between these proteins and ultrasonographic characteristics of the carotid plaque and more common drug usage in CAS patients. Previous study revealed that predominantly echolucent plaques compared with predominantly echogenic plaques were associated with an increased risk of stroke across all degrees of carotid stenosis (Kakkos et al., 2013; Brinjikji et al., 2015; Gupta et al., 2015). In this study, the results showed there were significant difference in expression level of interleukin-2 receptor subunit beta (IL2RB) between hypoechogenic plaque and other echogenic plaque, indicating IL2RB may involve in the pathophysiology of hypoechogenic plaque. CAS patients usually have diabetes or hypertension and managed with related drugs. We assessed potential influences of hypoglycemic drugs (acasbose and metformin) and hypotensive drugs (Dihydropyridine calcium antagonists: nifedipine or amlodipine, and Renin-angiotensin system inhibitors: angiotensin converting enzyme inhibitor or angiotensin II receptor blockers) on expression of urinary proteins. The data revealed that Serine/threonine-protein phosphatase PP1-gamma catalytic subunit (PPP1CC) was associated with the use of acasbose and lipoprotein lipase (LPL), and HLA class I histocompatibility antigen, B alpha chain (HLA-B) was connected to taking dihydropyridine calcium antagonists (*p* < 0.05). However, application of metformin and renin-angiotensin system inhibitors have no significant impact on expression of these DEPs.

### Diagnostic Performance of DEPs

**FIGURE 6:**
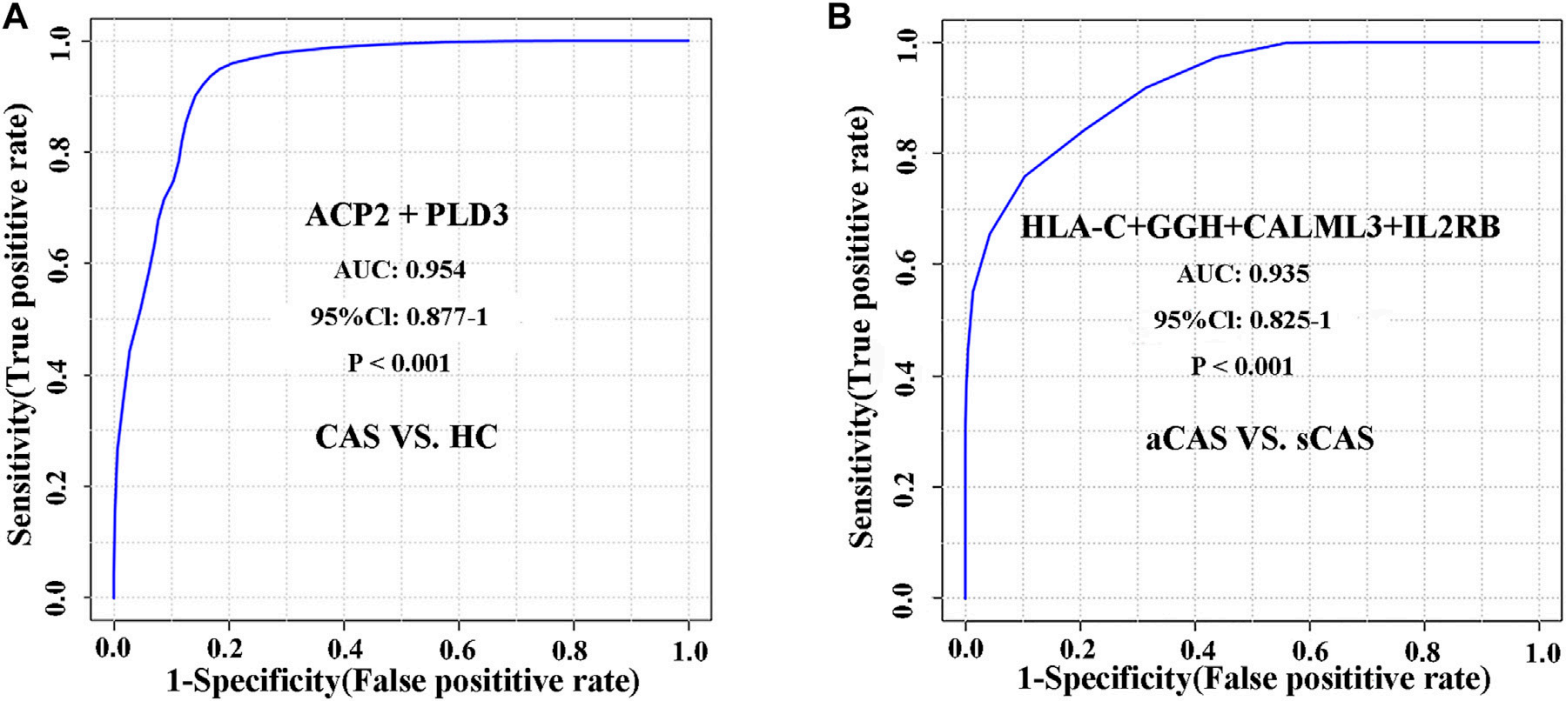
ROC analysis of differentially expressed proteins for distinguishing CAS patients and HCs and characterizing asymptomatic and symptomatic CAS patients. **(A)** CAS vs. HCs; **(B)** Asymptomatic CAS vs. symptomatic CAS.

To evaluate whether these candidate biomarkers could be used to diagnosis CAS, ROC analysis was performed (Table 2). Lysosomal acid phosphatase (ACP2) and 5′-3′ exonuclease (PLD3) independently showed excellent sensitivity and specificity for the diagnosis of CAS, with area under the curve (AUC) values of 0.933 and 0.924, respectively. When combined, the two proteins could achieve a higher diagnostic efficacy, with an AUC value of 0.954 (Figure 6A). The data showed that ACP2 and PLD3 could be used as potential diagnostic biomarkers of CAS.

**TABLE 2:** The results of ROC analysis for the potential biomarkers.

Uniprot Id	AUC	95% confidence interval	*p* Value	Sensitivity	Specificity	Positive predictive value (%)	Negative predictive value (%)
ACP2	0.933	0.830–0.983	<0.0001	88.57	88.89	93.9	80
PLD3	0.924	0.817- 0.979	<0.0001	80.00	94.44	96.6	70.8
HLA-C	0.822	0.656–0.930	<0.0001	100.00	69.57	63.2	100.0
GGH	0.815	0.648–0.926	<0.0001	75.00	82.61	69.2	86.4
CALML3	0.736	0.560- 0.870	0.0074	100	43.48	48.0	100.0
IL2RB	0.743	0.567–0.875	0.0048	91.67	56.52	52.4	92.9

ACP2, Lysosomal acid phosphatase; PLD3, 5′-3′ exonuclease; HLA-C, HLA class I histocompatibility antigen, C alpha chain; GGH, gamma-glutamyl hydrolase; CALML3, calmodulin-like protein 3; IL2RB, interleukin-2 receptor.

We further evaluated the diagnostic accuracy of several proteins to distinguish symptomatic and asymptomatic CAS. A protein panel consisting of HLA class I histocompatibility antigen, C alpha chain (HLA-C), gamma-glutamyl hydrolase (GGH), calmodulin-like protein 3 (CALML3) and IL2RB showed the best prediction accuracy, with an AUC of 0.935 (Figure 6B). The panel consisting of these urinary proteins could help with the risk stratification of ischemic stroke or TIA caused by CAS.

## Discussion

CAS is the principal cause of ischemic stroke. Therefore, the early diagnosis and risk stratification of CAS is a prime requirement for the determination of treatment plans and management of patients. At present, the diagnosis of CAS mainly depends on imaging (ultrasonography, magnetic resonance angiography, or computed tomography) (Gokaldas et al., 2015). However, these methods only can detect AS lesions that already formed, but can’t evaluate or predict the risk of stroke or TIA well. Besides, magnetic resonance angiography, or computed tomography is high cost or invasive. Novel blood biomarker for diagnosing CAS or predicting stroke has been widely studied, but they have not been applied in clinical practice due to sufficient accuracy (Martinez et al., 2020; Wijeratne et al., 2020). Moreover, the presence of high abundance proteins in the blood limits the detection of proteins with low abundance. Urine is an ideal source of biomarkers for clinical proteomics studies with many advantages such as noninvasive manner, large volume, easy collection, and stabilization. More importantly, without homeostatic control urine have the potential to detect the small and early pathological changes (Wu and Gao, 2015; Wu et al., 2021). Mass spectrum-based omics methods have been used in the biomarker discovery of CAS, and the sample source mainly contained blood or atherosclerosis plaque (Vorkas et al., 2016; Li et al., 2017; Lee et al., 2019; Ji et al., 2021). Until now, there is still no available urinary biomarker for CAS. Urinary proteomics research aimed at exploring the potential diagnosis and risk stratification biomarkers of CAS is necessary.

To the best of our knowledge, this is the first study to explore urinary biomarkers of CAS using the DIA-based proteomics strategy. A total of 194 urinary DEPs were identified between CAS patients and HCs. The signaling pathways involving these DEPs could characterize the pathophysiological changes in CAS patients. Among those DEPs, ACP2 and PLD3 both have a high AUC in predicting CAS. We further made multivariate regression analysis using age, sex, hypertension, diabetes and ACP2 or PLD3 as covariates respectively. The data showed ACP2 (odds ratio: 127.27; 95% confidence interval, CI: 10.676–1,517.184; *p* < 0.001) or PLD3 (odds ratio: 52.367; CI: 5.767–475.525; *p* < 0.001) was the independent predictors of CAS, suggesting a high diagnostic value. However, it may attribute to a relatively small sample that the range of CI was wide. A larger sample study is needed in the future. There is a much higher risk of ischemic stroke in symptomatic CAS patients than in asymptomatic patients. To identify potential biomarkers for the early prediction of stroke or TIA, we further compared urinary protein abundance between symptomatic and asymptomatic patients. A total of 32 DEPs were identified, and the signaling pathways associated with DEPs reflected the involvement of the immune/inflammatory response in symptomatic CAS. Finally, a panel of four urinary proteins (HLA-C + GGH + CALML3 + IL2RB) showed good discriminative power between symptomatic and asymptomatic CAS with an AUC value of 0.935.

In this study, IPA analysis of urinary differential proteins between CAS and HCs enriched pathways associated with CAS pathogenesis, including heme degradation, autophagy, coagulation system, IL-8 signaling, AS signaling, phospholipase C (PLC) signaling, and complement system pathways. These results were in accordance with the pathophysiological changes in atherosclerotic lesions. More interestingly, AS signaling was enriched in the CAS group, which also suggested the reliability of our proteomics results. The heme degradation process is mainly involved in the oxidation of heme by heme oxygenase-1 (HO-1). A previous study also showed that plasma HO-1 levels were higher in subjects with carotid plaques than in those without plaques (Kishimoto et al., 2018). Autophagy dysfunction can lead to vascular oxidative stress, an inflammatory response, and atherosclerotic plaque necrosis, which is closely associated with atherosclerotic development (Zhu et al., 2017). Moreover, autophagy pathways could contribute to the development of plaque vulnerability in the earlier stages of the atherogenic process of CAS (Magné et al., 2015). In addition, platelets and the coagulation system also play a significant role in atherogenesis (Páramo, 2011). Patients with CAS show activation of coagulation compared to HCs (Kölbel et al., 2010). Polymorphonuclear neutrophils from carotid plaques and peripheral blood of CAS patients can produce IL-8, which is crucial for atherosclerotic plaque development (Marino et al., 2015). PLC signaling can be initiated in macrophages, and the lack of PLC beta 3 could increase macrophages in atherosclerotic lesions and reduce the size of atherosclerotic lesions. Furthermore, PLC activity could promote macrophage survival in atherosclerotic plaques and contribute to AS, which suggests that PLC may be a potential target for the treatment of AS (Wang et al., 2008). The complement system is closely connected with AS and could be activated by cholesterol crystals (Niyonzima et al., 2017). Besides, the lectin pathway of complement activation in atherosclerotic plaques could also reflect the instability of the plaques (Fumagalli et al., 2017). Thus, urinary proteomics could reflect the pathophysiological process of CAS.

Pathways related to symptomatic CAS were also enriched by IPA analysis, including natural killer cell (NK) signaling, cytotoxic T lymphocyte-mediated apoptosis of target cells, phospholipases, IL-2 signaling, vascular endothelial growth factor (VEGF) signaling, and dendritic cell maturation. Previous studies showed that there was significantly more inflammatory cell infiltration, including NK and cytotoxic T cells, in symptomatic plaques than in asymptomatic plaques (Schumacher et al., 2001). Plaque destabilization in some CAS patients may be associated with NK signaling (Martínez-Rodríguez et al., 2013). Phospholipases are mainly secreted by immune cells and closely linked with inflammation and AS. Moreover, phospholipases are associated with the occurrence of cerebrovascular events in CAS patients with high stroke risk (Zhang et al., 2021). The IL-2 level was significantly higher in symptomatic patients than in asymptomatic CAS patients, indicating the potential predictive value of IL-2 signaling in symptomatic CAS (Stauss et al., 2020). In addition, VEGF is critical in the progression of carotid plaque and intraplaque hemorrhage. Symptomatic CAS has a higher expression level of VEGF in atherosclerotic plaques than asymptomatic CAS (Giannoni et al., 2009; Hiyama et al., 2010; Grufman et al., 2014; Basic et al., 2019). Moreover, dendritic cells are crucial regulators of the immune system and are associated with T cell differentiation. The numbers of total and mature dendritic cells were significantly higher in vulnerable carotid plaques than in stable plaques (Erbel et al., 2007; Dietel et al., 2013). In conclusion, most pathways are associated with immune/inflammatory pathways that could reflect the existence of a dramatic immune response and inflammation in the atherosclerotic lesions of symptomatic CAS compared to asymptomatic lesions.

Several potential protein biomarkers were identified in this study. ACP2 combined with PLD3 can separately distinguish CAS patients from HCs with an excellent AUC value. ACP2 is a ubiquitous lysosomal enzyme that catalyzes the hydrolysis of phosphate and is composed of alpha and beta subunits. The concerted action of ACP2 and ACP5 is required for dephosphorylation of mannose 6-phosphate-containing lysosomal proteins to promote the activity of specific lysosomal enzymes (Makrypidi et al., 2012; Pohl et al., 2018). Notably, in atherosclerotic plaques, macrophage lysosomes play a paramount role in dealing with an overabundance of lipids and cytotoxic material. The dysfunction of lysosomes in lipid degradation, macrophage polarization, generation of lipid signaling intermediates or mTOR activation can have profound effects on the development of atherosclerosis (Sergin et al., 2015). Thus, there are reasons to believe that ACP2 may be connected to atherosclerotic pathogenesis. Similar to the subcellular localization of ACP2, PLD3 is a lysosome-resident exonuclease with ssDNA as a substrate and is highly expressed in the brain (Gavin et al., 2018; Gonzalez et al., 2018). The function of PLD3 may be associated with cell differentiation, antiapoptosis, epigenetic modifications, and intracellular signal transduction (Wang et al., 2015). Furthermore, when combining PLD3 with previous IPA analysis, PLD3 was found to be involved in IL-8 signaling and phospholipase C signaling and may be associated with the pathophysiology of CAS. Clinically, PLD3 is closely related to Alzheimer’s disease, and it has been demonstrated to be involved in amyloid precursor protein processing in Alzheimer’s disease pathogenesis (Karch and Goate, 2015; Wang et al., 2015; Fazzari et al., 2017). Amyloid-beta (Aβ) is a proteolytic fragment of amyloid precursor protein. Interestingly, Aβ1-40 deposits have been found in carotid atherosclerotic plaques, and increased Αβ1-40 was related to higher carotid intima-media thickness (De Meyer et al., 2002; Stamatelopoulos et al., 2015; Bucerius et al., 2017). PLD3 may participate in the pathophysiological process of CAS.

Moreover, the combinations of HLA-C, GGH, CALML3 and IL2RB exhibited a higher AUC value in separating symptomatic CAS from asymptomatic CAS. HLA-C and IL2RB were found by IPA analysis to be involved in a series of immune/inflammatory pathways of symptomatic CAS, such as natural killer cell signaling, cytotoxic T lymphocyte-mediated apoptosis of target cells, antigen presentation pathway, T cell exhaustion signaling pathway, IL-2 signaling, and IL-15 signaling. There are more NK and cytotoxic T cells infiltration in symptomatic plaques than asymptomatic plaques (Schumacher et al., 2001). The major histocompatibility complex class I protein HLA-C plays an important role in delivering inhibitory signals to NK cells and cytotoxic T lymphocytes by killer cell Ig-like receptors (Zipeto and Beretta, 2012; Goodson-Gregg et al., 2020). Besides, IL2RB is expressed on T cells and NK cells and transfers IL-2 signals (Fernandez et al., 2019). Moreover, previous studies have revealed that high expression of the IL-2 signal was associated with symptomatic CAS (Stauss et al., 2020). GGH is involved in glutamate removal from folate signals in IPA analysis, and overexpression of GGH can lead to more efflux of folate, which might result in folate deficiency (Wani et al., 2012). Folate is related to the risk of ischemic stroke and cardiovascular disease (Weng et al., 2008; Luu et al., 2011; Wei et al., 2015). Additionally, the structure of calmodulin-like protein is similar to that of calmodulin, which can regulate signal transduction and metabolic activities related to calcium ion (Soderling et al., 2001). It has been reported that CALML3 are involved in cell proliferation and apoptosis (Bennett et al., 2013; Jia et al., 2018). Besides, previous studies have suggested that CALML3 may play critical roles in AS development mediated by icariin (Zhang et al., 2019).

In this study, the results suggested that the urinary proteome may be related to pathophysiological alterations in CAS, and we identified a protein panel that has a high value for diagnosing CAS and predicting early ischemic stroke or TIA. The results might contribute to the auxiliary clinical diagnosis and risk stratification of CAS. There are several limitations of this study. Firstly, it was performed at a single center and had a smaller sample size. Secondly, the biomarkers associated with symptomatic CAS are unable to predict cardio-embolic stroke, which represent a larger part of acute cerebrovascular events. Thirdly, this is a matched case-control study and some factors such as age, gender, comorbidities and drug usage may have influences on the results. Therefore, results interpretation should be with caution. Finally, the results lack of further validation with some other experimental techniques or a large sample. Nevertheless, this study is an exploratory study in which we utilized urine proteomics to attempt to discovery novel biomarkers of CAS and symptomatic CAS; to our knowledge, no study tried to search CAS biomarker from urine before this study. In the future, to verify the clinical application of these biomarkers, a large-sample, multicenter, prospective clinical validation study is needed.

## Conclusion

This is the first application of a DIA approach to research urinary protein biomarkers of CAS. The signaling pathways of the DEPs are consistent with the classic pathophysiological mechanism by which atherosclerotic CAS is closely related to substance/energy metabolism and the immune/inflammatory response. Furthermore, a panel consisting of six urinary proteins (ACP2, PLD3, HLA-C, GGH, CALML3 and IL2RB) has the potential to serve as diagnostic biomarkers or therapeutic targets of CAS and distinguish symptomatic from asymptomatic CAS well with the value of early prediction of ischemic stroke or TIA caused by CAS.

## Data Availability

The original contributions presented in the study are publicly available. This data can be found here: http://proteomecentral.proteomexchange.org.

## Supplementary Material

Supplementary Figure S1 Correlation analysis of mixed samples.

Supplementary Table S1 Identification details of the urine proteome in this study.

Supplementary Table S2 The DEPs between CAS patients and HCs.

Supplementary Table S3 The DEPs between asymptomatic and symptomatic CAS patients.
