# Supplementary figures and images for: Urinary Proteomics Identifying Novel Biomarkers for the Diagnosis and Phenotyping of Carotid Artery Stenosis
Source: Front Mol Biosci. 2021 Aug 10;8:714706. doi: 10.3389/fmolb.2021.714706 (PMC8383446; doi:10.3389/fmolb.2021.714706)

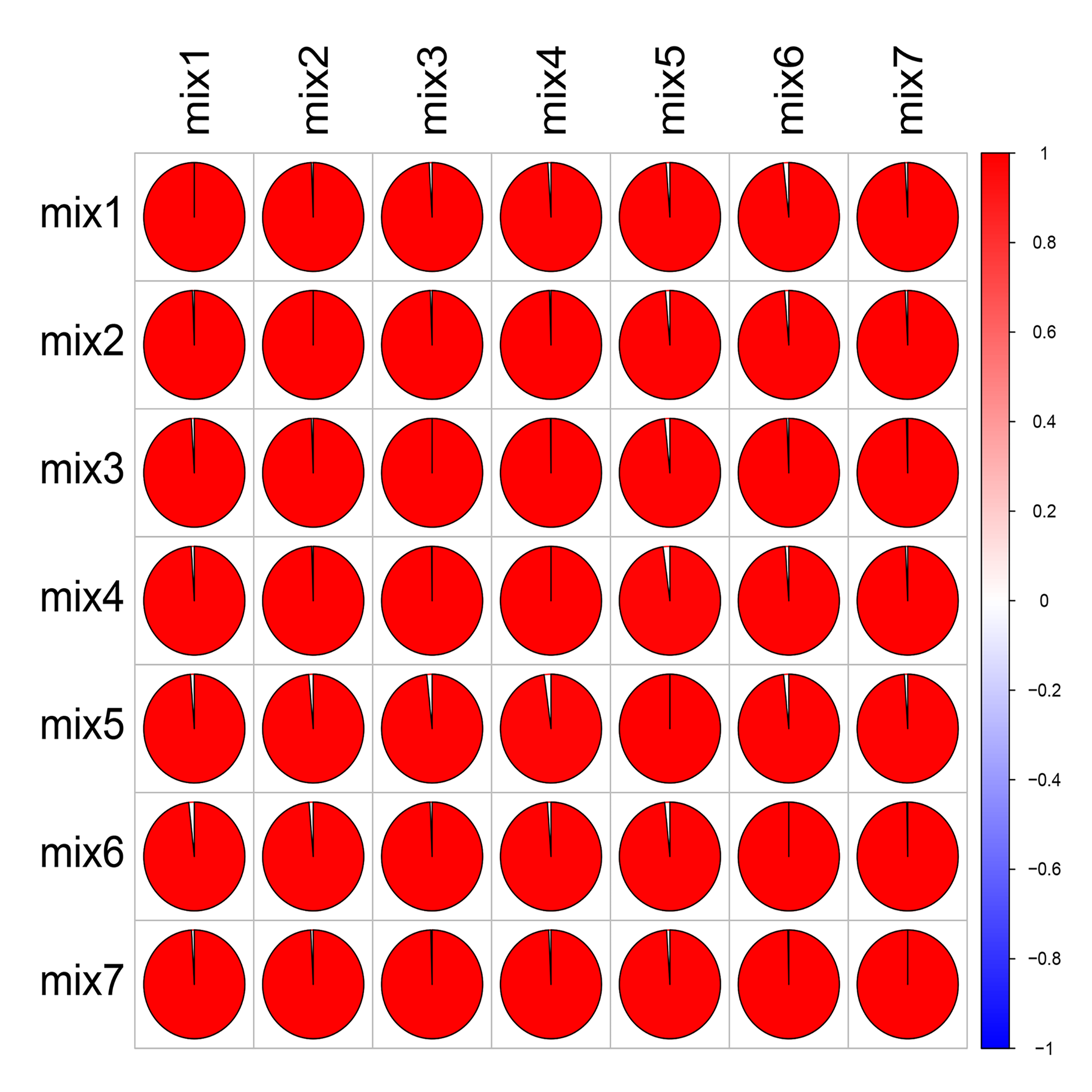

Supplement: Supplementary file 3 [file Image1.TIF]
